# Human Monocyte-Derived Dendritic Cells Are the Pharmacological Target of the Immunosuppressant Flavonoid Silibinin

**DOI:** 10.3390/ijms231810417

**Published:** 2022-09-08

**Authors:** Maria Teresa Pagano, Katia Fecchi, Marina Pierdominici, Elena Ortona, Daniela Peruzzu

**Affiliations:** Center for Gender Specific Medicine, Istituto Superiore di Sanità, 00161 Rome, Italy

**Keywords:** dendritic cells, autoimmunity, immunosuppression

## Abstract

Silibinin, a natural polyphenolic flavonoid, is known to possess anti-inflammatory, anticancer, antioxidant, and immunomodulatory properties. However, the effects of Silibinin on the maturation and immunostimulatory functions of human dendritic cells (DC) remain to be elucidated. In this study, we have attempted to ascertain whether Silibinin influences the maturation, cytokine production, and antigen-presenting capacity of human monocyte-derived DC. We show that Silibinin significantly suppresses the upregulation of costimulatory and MHC molecules in LPS-stimulated mature DC and inhibits lipopolysaccharide (LPS)-induced interleukin (IL)-12, IL-23, and TNF-α production. Furthermore, Silibinin impairs the proliferation response of the allogenic memory CD4 T lymphocytes elicited by LPS-matured DC and their Th1/Th17 profile. These findings demonstrate that Silibinin displays immunosuppressive activity by inhibiting the maturation and activation of human DC and support its potential application of adjuvant therapy in the treatment of autoimmune diseases.

## 1. Introduction

Silibinin, a natural polyphenolic flavonoid, is the major biologically active component of Silymarin isolated from the plant milk thistle Silybum marianum. Silibinin has been shown to have multiple protective biological activities due to its ability to modulate various cellular signaling pathways and to possess strong antioxidant, hepatoprotective, neuroprotective, anticancer, and antiviral activities [1]. It has been traditionally used for the treatment of various liver disorders [2] as well as of neurodegenerative diseases, including Alzheimer’s disease, Parkinson’s disease, and depression, due to its ability to increase the intracellular antioxidant defense and restore the redox status by changing the level of antioxidant enzymes [3]. The induction of apoptosis and its other abilities including cell growth inhibition, cell cycle arrest, an antiproliferative effect, and regulation of aberrant miRNA expression have supported the use of Silibinin as an anticancer and chemo preventive agent in a variety of cancers [4].

The biological actions of Silibinin include a broad spectrum of anti-inflammatory and immunosuppressive effects in a dose and time-dependent manner [5]. The potent anti-inflammatory effects of Silibinin have been shown to be related to the inhibition of several transcriptional factors such as the Nuclear Factor kappa B (NF-κB) and the Signal Transducer and Activator of Transcription 3 (STAT-3) which regulate several gene products involved in the immune response, inflammation, and cytokine production [6,7]. Silibinin is known to inhibit CD4^+^ T-cell activation and proliferation by inhibiting the Mitogen-activated protein (MAP) kinase pathway through T-cell receptor engagement [8] and to suppress the production of Th1-related cytokines (IL-2, IFN-γ, and TNF-α) by activated-PBMC from HCV-infected and uninfected subjects [9].

In agreement with these studies, we previously showed that Silibinin upregulates ERβ expression, induces apoptosis, inhibits proliferation, and reduces expression of the pro-inflammatory cytokines through ERβ binding in T lymphocytes from female and male healthy donors and patients with active Rheumatoid Arthritis [10]. Moreover, Silibinin has been also shown immunosuppressive effects in experimental autoimmune encephalomyelitis (EAE), the animal model of multiple sclerosis via inhibition of dendritic cell (DC) activation, and Th17 cell differentiation [11]. Silibinin has also been demonstrated to inhibit phenotypic and functional maturation of murine bone marrow-derived DC and Th1 polarization. In particular, it has been shown that Silibinin suppresses the expression of MHC class I and II and costimulatory molecules (CD80, CD86) and IL-12 production by murine bone marrow-derived DC [12].

DC play a key role in both the pathogenesis and treatment of several diseases. Therefore, DC are attractive targets for therapeutic manipulations of the immune response. However, the effect of Silibinin on human DC has never been investigated. Here, we show that Silibinin affects the LPS-induced maturation of human monocyte-derived DC by inhibiting the upregulation of costimulatory and MHC molecules and inflammatory cytokine production. Moreover, Silibinin treated DC fail to present alloantigens to memory CD4^+^ T lymphocytes which barely proliferate and the percentage of IFN-γ^+^ and IL-17^+^ CD4^+^ T-cells is significantly lower.

Given the critical role of DC in the initiation and regulation of immune responses, these findings provide new insight into the immunosuppressive activity of Silibinin and support its potential application in the treatment of autoimmune diseases.

## 2. Results and Discussion 

### 2.1. Silibinin Inhibits the Phenotypical Maturation of DC Induced by LPS Stimulation

DC play a pivotal role in the control of the innate and adaptive immune response. They reside in peripheral tissues and undergo a maturation process in response to diverse stimuli. Mature DC express high levels of major histocompatibility complex (MHC) molecules and co-stimulatory molecules that are important for antigen presentation to T lymphocytes.

**Figure 1:**
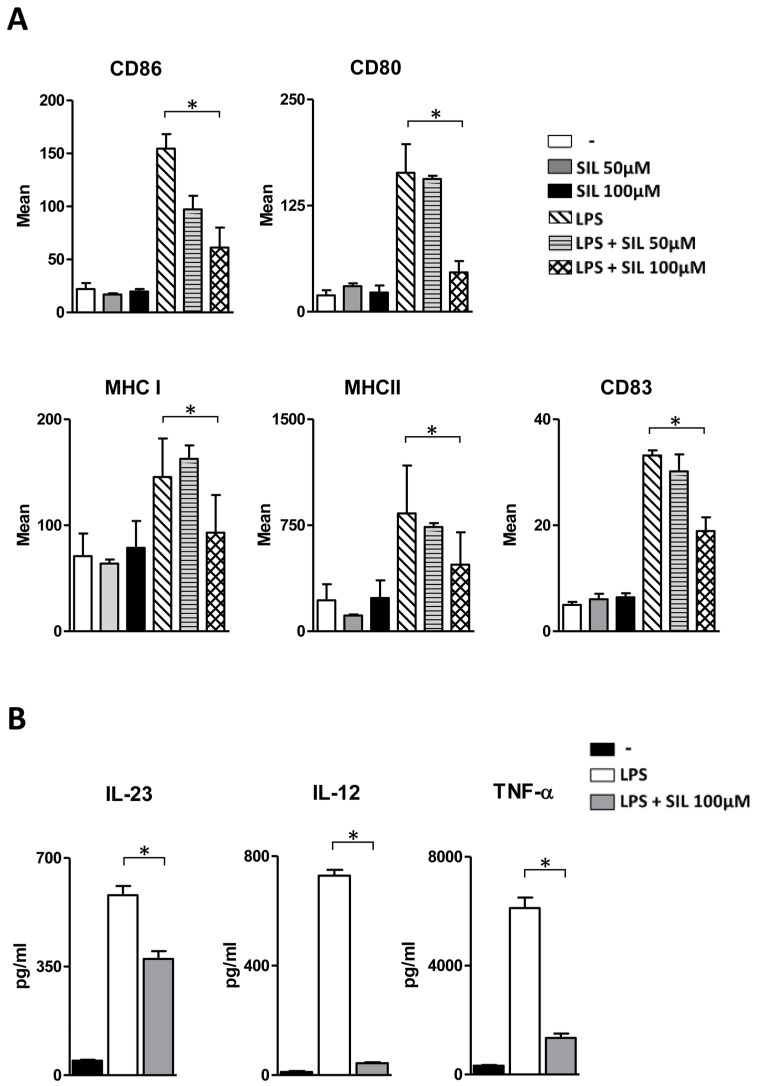
Silibinin significantly suppresses the upregulation of costimulatory and MHC molecules and cytokine production during the maturation process of DC. (**A**) Human monocyte-derived DC were cultured for five days in IL-4 and GM-CSF-enriched medium and stimulated overnight with LPS in the presence or absence of Silibinin at different concentrations (50 or 100 µM). LPS-stimulated DC were used as positive controls for DC maturation. The expression of surface molecules (CD86, CD80, CD83, MHC I, and MHC II) was analyzed using flow cytometry and represented as histograms with the mean fluorescence intensity. Results are expressed as the mean ± standard deviation (SD) of three independent experiments performed. (**B**) Human monocyte-derived DC were cultured for five days in IL-4 and GM-CSF-enriched medium and stimulated overnight with LPS in the presence or absence of Silibinin at 100 µM. Culture supernatants were collected after 24 h and the levels of the proinflammatory cytokines IL-23, IL-12, and TNF-α were determined by ELISA. Results are expressed as the mean ± SD of three independent experiments performed. Statistical analysis was performed using GraphPad Prism (version 5.0, GraphPad Software, San Diego, CA, USA).* *p* < 0.05 was considered statistically significant.

To investigate the effect of Silibinin on DC, phenotype cells were cultured for five days in IL-4 and GM-CSF-enriched medium and stimulated overnight with LPS in the presence or absence of Silibinin at different concentrations (50 or 100 µM). Figure 1A shows that Silibinin added on the fifth day of culture did not alter the DC phenotype at either concentration. When added together with LPS at 50 µM, it inhibited only the upregulation of CD86 costimulatory molecule, whereas at 100 µM, it significantly suppressed the up-regulation of co-stimulatory molecules CD80 and CD86 and of MHC class I and II induced by LPS stimulation. Furthermore, Silibinin decreased the novo-expression of CD83, a maturation marker for DC. These data indicate that Silibinin alters the DC maturation program which is needed for acquiring a fully professional antigen-presenting cell profile.

### 2.2. Silibinin Inhibits Proinflammatory Cytokine Production by LPS-Stimulated DC

The maturation process of DC induced by several stimuli leads to the production of a cascade of pro-inflammatory cytokines, which in turn control the induction and skewing of T-cell responses. LPS-stimulated DC produce huge amounts of IL-12 and IL-23 which are involved in Th1 and Th17 polarization, respectively, and of TNF-α cytokine which has pleiotropic effects on various cell types and is a major regulator of inflammatory responses. To investigate the effects of Silibinin on the pro-inflammatory cytokine profile of mature DC, we measured IL-12, TNF-α, and IL-23 secretion by DC stimulated with LPS in the presence of the flavonoid. Figure 1B shows a significant decrease in all three cytokines induced by a Silibinin treatment at 100 µM, thereby indicating an anti-inflammatory effect of Silibinin.

### 2.3. Mature DC Treated with Silibinin Do Not Present Alloantigens to Memory CD4^+^ T Lymphocytes

**Figure 2:**
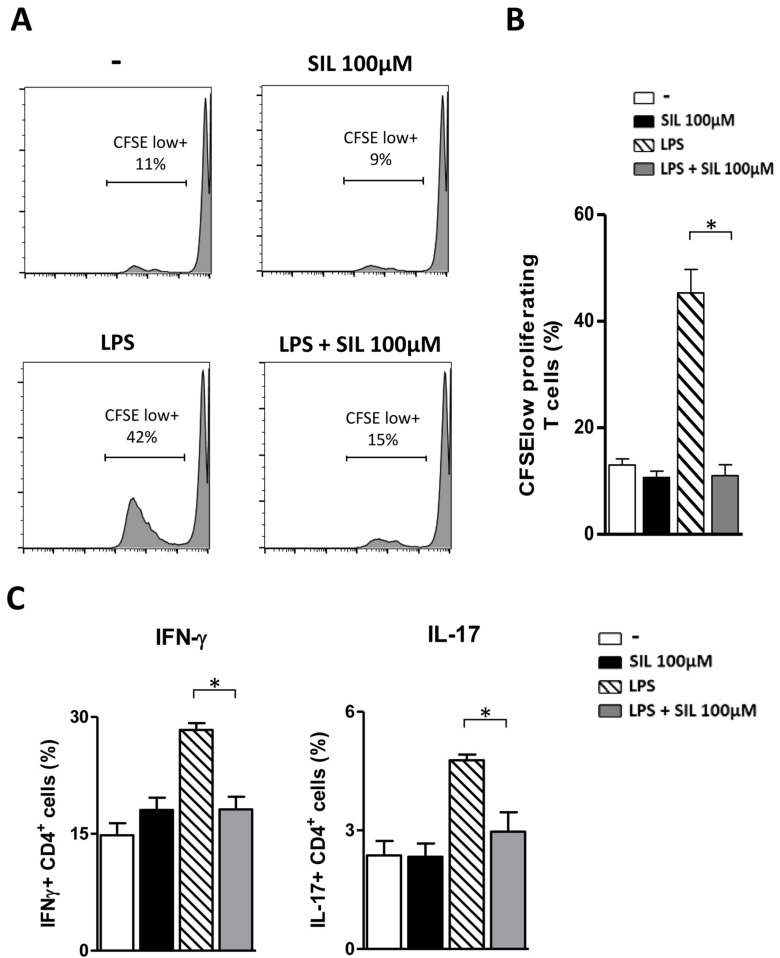
Silibinin impairs the allo-stimulatory ability of LPS-stimulated DC on memory CD4^+^ T lymphocytes. Human monocyte-derived DC were cultured for five days in IL-4 and GM-CSF-enriched medium and incubated for 24 h with medium alone, Silibinin (100 µM), LPS (100 ng/mL), or Silibinin with LPS. On day 6, a mixed leukocyte reaction (MLR) was performed. The DC were co-cultured with memory CD4^+^ T lymphocytes labeled with CFSE at a ratio of 1:10. (**A**) CD4^+^ T-cell proliferation was analyzed by flow cytometry after 7 days. The numbers in the histograms represent the percentage of dividing cells (CFSE low^+^). The data are representative of three independent experiments performed. (**B**) Tests were conducted in triplicate and the results are expressed as the mean ± SD. (**C**) CD4^+^ cells were examined via intracellular cytokine staining. The percentage of intracellular IFN-γ^+^ and IL-17^+^ CD4^+^ T-cells was determined by flow cytometry as described in Section 3. Results are expressed as the mean ± SD of three independent experiments performed. Statistical analysis was performed using GraphPad Prism Software v.5. * *p* < 0.05 was considered statistically significant.

To investigate the effects of Silibinin on the antigen-presenting capacity of DC, we measured CD4^+^ T-cell response to alloantigens in a mixed leukocyte reaction. As shown in Figure 2, LPS-matured DC, as expected, induced a strong proliferation of allogeneic CD4^+^ T-cells and an increase in the percentage of IFN-γ and IL-17 producing CD4^+^ T-cells as compared to immature or Silibinin-treated DC. On the contrary, the same population of T-cells barely proliferated and the percentage of IFN-γ and IL-17 CD4^+^ T-cells was significantly lower in response to LPS-matured DC treated with Silibinin. These results demonstrate the immunosuppressive action of Silibinin on the most powerful antigen-presenting cells which play a crucial role in shaping the immune response in several diseases.

## 3. Materials and Methods

### 3.1. Monocyte Isolation and DC Generation

Peripheral blood mononuclear cells (PBMC) were purified from heparinized blood obtained by healthy donors on a density gradient (Ficoll-Hypaque, Lympholyte-H; Cedarlane, Paletta Court, ON, Canada).

Monocytes were purified by positive sorting using anti-CD14-labeled magnetic microbeads (Miltenyi Biotec, Bergisch Gladbach, Germany) according to the manufacturer’s instructions. DC were generated by culturing monocytes in 6-well tissue culture plates (Corning Costar, Camelback Rd., Glendale, AZ, USA) with 50 ng/mL granulocyte-macrophage colony-stimulating factor (GM-CSF; Gentaur, Bergamo, BG, Italy) and 20 ng/mL of IL-4 (R&D Systems, Minneapolis, MN, USA) for 5 days at a concentration of 4 × 105 cells/mL in RPMI 1640 supplemented with 2 mML-glutamine, 1% nonessential amino acids, 1% sodium pyruvate, 50 µg/mL kanamycin (Gibco, Billings, MT, USA), and 10% fetal bovine serum FBS (Corning). On day 5, the culture medium containing IL-4 and GM-CSF was replaced with the culture medium alone, 20 h before treatments.

### 3.2. Abs and Other Reagents

For surface staining, fluorescein isothiocyanate (FITC) or R-phycoerythrin (PE) conjugated monoclonal antibodies (mAbs) specific for CD80, CD86, CD83, MHC I, and MHC II (Miltenyi) were used. Lipopolysaccharide (LPS) from *Escherichia coli* 0111:B4 (Sigma-Aldrich, St. Louis, MO, USA) was used at a concentration of 100 ng/mL to induce DC maturation.

Silibinin (Sigma, St. Louis, MO, USA) was dissolved in dimethyl sulfoxide, diluted in the culture medium, and used at 50 or 100 µM.

### 3.3. Flow Cytometric Analysis

Immature DC or LPS-stimulated DC in the presence or absence of Silibinin at 50 or 100 µM were plated into 96-well plates and washed once in phosphate-buffered saline (PBS) containing 1% FBS. The cells were incubated with Mabs at 4 °C for 30 min. Cells were then analyzed on a Beckman Coulter Gallios™ flow cytometer and data were analyzed with Kaluza Analysis Software v2.1 (Beckman Coulter, Brea, CA, USA).

### 3.4. Cytokine Production Analysis

After 5 days, the supernatant of DC cultures was removed, and the cells were washed and cultured in the presence or absence of 100 ng/mL LPS or Silibinin 100 µM for an additional 24 h. After 5 days, the supernatants from immature DC or LPS-stimulated DC cultures in the presence or absence of 100 µM Silibinin were harvested, filtered (0.2-μm filters), and stored at −80 °C. IL-23, IL-12p70, and TNF-α were measured by the specific enzyme-linked immunosorbent assay (ELISA) using the commercially available kit (R&D Systems Europe, Ltd., Abingdon, UK) according to the manufacturer’s instructions.

### 3.5. Mixed Leukocyte Reaction (MLR)

Immature DC or LPS-stimulated DC were or were not treated with Silibinin (100 µM), washed with PBS, and then co-cultured with CD4^+^ lymphocytes purified by indirect magnetic sorting with a memory CD4^+^ T-cell isolation kit (Miltenyi) at 1:10 ratio. To track proliferating cells, CD4^+^ T-cells were intracellularly labeled with the fluorescence dye carboxyfluorescein succinimidile estere (CFSE) (Thermo Fisher Scientific, Waltham, MA, USA) which decreases after each cell division. The proliferative response was evaluated after 7 days by measuring the CFSE dilution by flow cytometry and identified by the percentage of CFSE low^+^ cells. CFSE-unlabeled CD4^+^ T-cells were also analyzed by flow cytometry for their intracellular cytokine production.

### 3.6. Analysis of Intracellular Cytokine Production

CD4^+^ T-cells after stimulation with 50 ng/mL phorbol myristate acetate (PMA) and 1μg/mL ionomycin (both from Sigma) for 5 h and in the presence of Golgi Plug (BD Bioscience, San Jose, CA, USA) for the last 2 h of culture, were stained with FITC conjugated anti-human IL-17A Mab and APC/Cyanine7 conjugated anti-human IFN-γ Mab (BD Bioscience PharMingen) after fixation and permeabilization using Cytofix/Cytoperm (BD Bioscience PharMingen), according to the manufacturer’s instructions. Stained cells were analyzed on a Beckman Coulter Gallios™ flow cytometer and data were analyzed with Kaluza Analysis Software v2.1 (Beckman Coulter).

### 3.7. Statistical Analysis

Statistical analysis was performed by the Mann–Whitney U test using GraphPad Prism (version 5.0, GraphPad Software, San Diego, CA, USA). A *p*-value < 0.05 was considered statistically significant.

## 4. Conclusions

Dendritic cells are specialized antigen-presenting cells that maintain immune tolerance to self-antigens by deleting or monitoring auto-reactive T-cells. Several immunosuppressive and anti-inflammatory agents interfere with phenotypic and functional DC maturation, a classical example of which is represented by corticosteroids [13]. Our data demonstrate that the flavonoid Silibinin has immunosuppressive and anti-inflammatory effects by hampering the maturation, inflammatory cytokine production, and antigen-presenting capacity of human monocyte-derived DC. Such evidence encourages the use of Silibinin as a therapeutic tool or a dietary nutritional supplement for the treatment of inflammatory/autoimmune diseases. However, further investigations in vivo are needed to support Silibinin application and therapeutic use in regard to its biological activities.

## Data Availability

The data supporting this study’s findings are available upon request.
